# COVID-19 Pharmacotherapy in Pregnancy: A Literature Review of Current Therapeutic Choices

**DOI:** 10.3390/v15030787

**Published:** 2023-03-19

**Authors:** Karolina Akinosoglou, Georgios Schinas, Emmanouil-Angelos Rigopoulos, Eleni Polyzou, Argyrios Tzouvelekis, George Adonakis, Charalambos Gogos

**Affiliations:** 1 Department of Medicine, University of Patras, 26504 Patras, Greece; 2 Department of Infectious Diseases, University General Hospital of Patras, 26504 Patras, Greece; 3 Division of Internal Medicine, University General Hospital of Patras, 26504 Patras, Greece; 4 Department of Pulmonology, University General Hospital of Patras, 26504 Patras, Greece; 5 Department of Obstetrics and Gynecology, University General Hospital of Patras, 26504 Patras, Greece

**Keywords:** SARS-CoV-2, COVID-19, pregnancy, remdesivir, nirmatrelvir/ritonavir, tixagevimab/cilgavimab, tocilizumab, anakinra, dexamethasone, baricitinib

## Abstract

The clinical management of COVID-19 in pregnant women, who are considered a vulnerable population, remains uncertain even as the pandemic subsides. SARS-CoV-2 affects pregnant individuals in multiple ways and has been associated with severe maternal morbidity and mortality, as well as neonatal complications. The unique anatomy and physiology of gestation make managing COVID-19 in this population a complex and challenging task, emphasizing the importance of spreading knowledge and expertise in this area. Therapeutic interventions require distinct clinical consideration, taking into account differences in pharmacokinetics, vertical transmission, drug toxicities, and postnatal care. Currently, there is limited data on antiviral and immunomodulating COVID-19 pharmacotherapy in pregnancy. Some medication has been shown to be safe and well tolerated among pregnant women with COVID-19; however, the lack of randomized clinical trials and studies in this patient population is evident. Available vaccines are considered safe and effective, with no evidence of harm to the fetus, embryo development, or short-term postnatal development. Pregnant women should be counseled about the risks of SARS-CoV-2 infection and informed of available ways to protect themselves and their families. Effective treatments for COVID-19 should not be withheld from pregnant individuals, and more research is needed to ensure the best outcomes.

## 1. Introduction

The pathogenetic nature of SARS-CoV-2 and the ensuing COVID-19 pandemic has caused much uncertainty regarding the clinical management and potential outcomes of the disease in special populations. Pregnant women were among the first to be of concern. Pregnancy is a unique physiologic condition that may constitute a state of vulnerability to systematic infection. In particular, adaptive immune system changes that facilitate the induction and preservation of pregnancy may induce increased susceptibility to infection and may account for some of the severe presentations of the disease among pregnant populations.

COVID-19, being a multisystem disease, affects pregnant women and their bearing in multiple ways and on different levels [1]. Nonetheless, much of the current research focuses on the imminent result of respiratory compromise caused by the SARS-CoV-2 virus. Severe acute respiratory syndrome (SARS) is a precarious condition for pregnant populations that has been shown to pose a significant risk to both the mother and fetus [2]. COVID-19 in pregnancy was associated with consistent and substantial increases in severe maternal morbidity and mortality and neonatal complications when compared to pregnant women without COVID-19 [3]. Risk factors for severe maternal morbidity in pregnancy may also increase risk of COVID-19 illness in pregnancy [4].

COVID-19 management has rapidly evolved during the last three years, including a variety of components in the context of required antiviral, as well as antiinflammatory, therapy. Many of the initially adopted regimens were quickly abandoned in the absence of sufficient evidence, lack of proven benefit, or apparent toxicity. However, undoubtedly, current curative approaches bear a significant impact in reducing the need for hospitalization and mortality [5]. At the moment, according to living guidelines, therapeutic management of nonhospitalized individuals with COVID-19 includes the use of nirmatrelvir/ritonavir p.o (AII). or i.v. remdesivir (BII) for patients at high risk of severe disease, while when those are unavailable, the use of molnupiravir (CII) is recommended [6]. In case of hospitalized adults and/or severe disease, combination with antiinflammatory regimens including systemic corticosteroids, tocilizumab (antagonist of the interleukin-6 receptor), or baricitinib (Janus kinase inhibitor) is supported [6]. Certain groups of patients may benefit from the use of anakinra (interleukin-1 receptor antagonist). Prophylaxis is mainly achieved by vaccination, while until recently the use of monoclonal anti-SARS-CoV-2 antibodies, i.e., tixagevimab/cilgavimab, was recommended in populations with inadequate immunologic response [6,7].

Therapeutic interventions in pregnancy require distinct clinical management, given the organic specificities concerning the mother and fetus. Even though guidelines pertaining to therapeutic regimes do not differ between pregnant and nonpregnant populations [6], a number of issues need to be taken into consideration. Fetal exposure and potential adverse effects including congenital anomalies, preterm birth, and low birth weight remain of concern. Drug interaction with pregnancy comedications leads to increased or decreased exposure to certain components. Distinct differences in pharmacokinetics and pharmacodynamics occur in pregnant individuals compared to the general population. During pregnancy, changes in blood flow, body fluid volumes, and organ size can affect the pharmacokinetics of different regimens. For example, increased blood flow to the kidneys during pregnancy can increase the elimination of some drugs. Conversely, a decrease in gastrointestinal motility can delay the absorption, leading to higher drug concentrations in the blood. Similarly, exposure during lactation and respective variations in breast tissue and milk composition can pose different impacts of COVID-19 pharmacotherapy on an infant subject to lactation. These are only a few issues among those that caregivers have to take into account in the management of COVID-19 pregnant women. In this context, the decreased functional residual capacity (FRC) of a pregnant woman’s lungs also requires meticulous and careful consideration of interventional therapeutic modalities in order to maintain the increased arterial partial concentration of oxygen (PaO2) required to sustain the pregnancy and ensure the well-being of the fetus.

All in all, the anatomic and physiologic alterations caused during the pregnancy set the scene for a complex and constantly changing pathophysiologic background, which may complicate outcomes and exalt disease severity; thus, disseminating knowledge and expertise on the matter is of pivotal importance to ensure the development of obstetric COVID-19 management and help protect women’s lives everywhere. Currently, definitive answers concerning the effect of COVID-19 on pregnancy and vice versa have only recently begun to emerge.

We aimed to provide a literature review of available data concerning antiviral and immunomodulating COVID-19 pharmacotherapy in pregnancy and issues to be considered.

## 2. Materials and Methods

**Figure 1:**
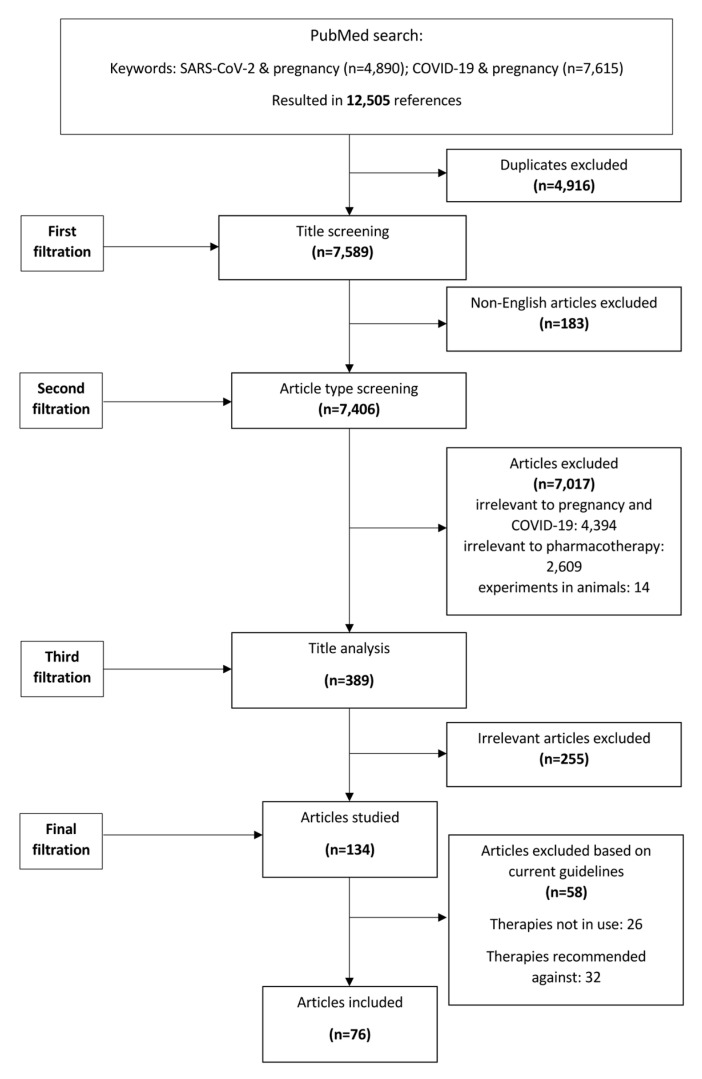
Study flowchart.

We performed a literature review following extensive searches of Scopus, Pubmed, and Embase by using the terms “COVID-19 AND pregnancy”, “SARS-CoV-2 AND pregnancy” between 1 January 2020 and 1 January 2023. Four independent reviewers reviewed articles and hand-searched literature, putting focus on antiviral and/or immunomodulating pharmacotherapy. Only English-language papers were reviewed. Regimens chosen to be reported were limited to those currently recommended in COVID-19 management guidelines, as per living guidance [6]. The main focus was put on human data. Regimens recommended against, as well as regimens pertaining to, supportive care (e.g., anticoagulants, bronchodilators, etc.) were not recorded in this review. Irrelevant articles, as well as duplicates, were removed, and disagreements were discussed and resolved. A study flowchart is shown in Figure 1.

## 3. Discussion

### 3.1. Pharmacotherapy

**Figure 2:**
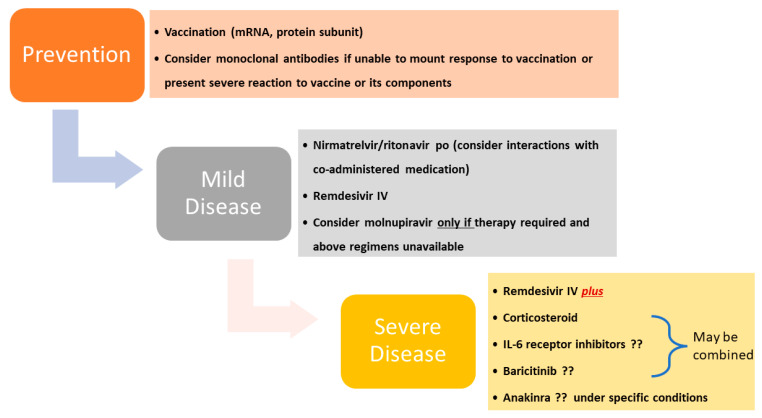
Recommended therapeutic regimens in pregnancy according to NIH living guidance. “?” indicates uncertainty or lack of evidence.

Most issues related to COVID-19 and therapeutic management do not seem to differ between pregnant and nonpregnant patients, and conform to current guidelines (Figure 2) [6]. However, a number of exceptions exist in the setting of pharmacologic management that dictates consideration of differences in pharmacokinetics and volume distribution, vertical transmission, and drug toxicities and postnatal care, including lactation.

### 3.2. Antivirals

#### 3.2.1. Remdesivir

In the setting of uneasiness in the performance of clinical trials among the pregnant population, experience on therapy has come from the compassionate use program [8]. Initially, the largest report came from the Gilead Global Safety Database (*n* = 156), which included pregnant patients with Ebola virus infection and COVID-19 [9]. Remdesivir administration took place after the first trimester in all cases Among this cohort, there were 33 live births, seven spontaneous fetal losses, two induced abortions, and four stillbirths. Five cases of congenital abnormalities were detected [9]. Among individuals that experienced adverse pregnancy outcomes, all were critically ill and required immediate invasive mechanical ventilation [9]. Compassionate use of remdesivir among 86 pregnant women hospitalized with COVID-19 between 21 March 2020 and 16 June 2020 showed a high rate of recovery, while RDV demonstrated no new safety signals [8]. This comes in line from data from a Phase IV prospective, open-label, nonrandomized, opportunistic pharmacokinetic study showing remdesivir was safe and well tolerated among pregnant women [10]. Among the 26 women who were pregnant at the time of RDV initiation, no obstetric indications for preterm delivery were reported, while among the 45 deliveries (including 19 postpartum), no neonatal deaths during the observation period were noted [8]. Cesarean delivery rate was higher among women who received remdesivir postpartum vs. during pregnancy [8]. Early RDV administration was associated with improved clinical outcomes, including lower rates of ICU admission and decreased progression to critical disease in pregnant individuals hospitalized with COVID-19 [11], in line with reports in the general population [12]. Similarly, early RDV administration was associated with improved pregnancy outcomes, including numerically lower rates of preterm delivery and maternal death from COVID-19 in pregnant individuals hospitalized with COVID-19 [11]. Data from 35 hospitalized pregnant women during the period from 1 April to 31 December 2020 were retrospectively analyzed, and the results showed that all 17 women who initiated RDV within 48 h from admission presented prompt improvement and recovered by day 7 [13]. No adverse findings have been observed in preclinical studies [14]. Prodrug remdesivir is unlikely to cross the placenta, in contrast to its circulating active metabolites, which have long half-lives, low molecular weights, and high unbound fractions [15].

Data pertaining to the impact of remdesivir on lactation are lacking. However, infants are unlikely to absorb clinically significant amounts from breastfeeding, since remdesivir has poor oral bioavailability [9]. Even in the case of potential systemic metabolite exposure, reports as to safety have been reassuring [16,17].

#### 3.2.2. Nirmatrelvir/Ritonavir

Use of nirmatrelvir/ritonavir is considered safe in pregnancy, with the data also remaining very limited [18]. Experience of outpatient treatment of seven women resulted in good health and no perinatal adverse outcomes [18]. Similarly, in a case series of 47 pregnant patients, nirmatrelvir/ritonavir was tolerated well by themselves and no adverse events on the offspring were recorded, even though an unexpected rate of cesarean section was noted [19]. In the paucity of data, but on the knowledge of the mechanisms of action for both agents and evidence from animal studies, the American College of Obstetricians and Gynecologists and the Society for Maternal–Fetal Medicine advocated for pregnant people’s access to nirmatrelvir/ritonavir since the potential benefits likely outweigh the risks [20], in line with NIH living guidance [6]. Limited data suggest that ritonavir is present in breast milk, but no information is available on the effects on the breastfed infant or the effects on milk production. Lactation is not a contraindication to use. If nirmatrelvir is required by the mother, it is not a reason to discontinue breastfeeding, but until more data are available, the infant should be monitored for adverse effects.

#### 3.2.3. Molnupiravir

Molnupiravir is a mutagenic ribonucleoside antiviral agent that has shown antiviral activity against SARS-CoV-2 both in vitro as well as in clinical trials [21,22]. Even though it has a lower efficacy than other approved antiviral agents, it remains an option when remdesivir or nirmatrelvir/ritonavir are not available. However, as a mutagenic agent it runs the theoretical risk of incorporating into the host DNA, leading to mutations. Experimental results in rodents—requiring 7.5 times more exposure than the human dosage to result in teratogenicity and embryo–fetal—in combination with brief duration of administration, led the FDA to conclude that the regimen has a low potential of genotoxicity [23]. However, living guidance recommends against its use in pregnancy, except when there is a lack of alternatives [6].

### 3.3. Immunomodulatory Therapy

#### 3.3.1. Corticosteroids

Dexamethasone was shown to decrease mortality in individuals with COVID-19 who require oxygen therapy, and was thus initially included in international recommendations [6,24]. Nonetheless, from the trials that followed, only in REMAP-CAP (number of pregnant patients not reported) [25] and RECOVERY (*n* = 4, 0.06% of participants) were pregnant patients included [24]. Even though dexamethasone’s benefit may be attributed to class effect, hydrocortisone use has been less effective [26]. During pregnancy, the choice of corticosteroid has traditionally depended upon whether treatment was intended for the mother or the fetus. In the former case, hydrocortisone, methyl-prednisolone, prednisolone, and prednisone that is metabolized to inactive metabolites by placental enzymes and hence limits fetal exposure to 10% of maternal intake are preferred [27,28]. In the latter case, when treatment for the fetus is commonly used to promote fetal lung maturation, synthetic fluorinated corticosteroids, such as dexamethasone and betamethasone are chosen, in combination with their minimal mineralocorticoid activity [27,29]. After the initial four doses of dexamethasone, some guidelines switch to hydrocortisone or methylprednisolone to minimize fetal glucocorticoid exposure beyond the standard used for fetal lung maturation [30]. Associations with corticosteroid use and adverse pregnancy outcomes, including congenital malformations, intrauterine growth restriction, gestational diabetes, preterm birth, and preeclampsia have been variably reported in the past [28,31]. However, assessing corticosteroid—and dexamethasone in particular—safety in this setting remains challenging, since multiple cofounders, including comedications and overrepresentation of high-risk pregnancies in these studies, introduce an inevitable bias [28]. Glucose levels should be monitored closely in patients with gestational or pregestational diabetes, as hyperglycemia will usually occur after administration of dexamethasone. Amounts of dexamethasone in breast milk is low and is to be considered safe if used for short durations during lactation, even though high-dose corticosteroids may temporarily decrease milk supply [32].

#### 3.3.2. Tocilizumab

Tocilizumab represents a recombinant humanized monoclonal IgG1 antibody against interleukin-6 (IL-6) receptor. Tocilizumab seems to reduce mortality in hospitalized adults with severe or critical COVID-19, even though current data remain conflicting [24,33]. Transplacental transport of IgG is thought to be very low during the first trimester of pregnancy, but it increases steadily thereafter [34]. As a result, during the critical period of organogenesis, fetal exposure is likely minimal [34,35]. Clinical data on tocilizumab use in pregnant women mostly derive from the Roche Global Safety Database [36] and the European League against Rheumatism (EULAR) task force [37]. The former database included over 90% cases with rheumatoid arthritis, receiving their last dose preconception or early; hence, no drug was expected to cross the placenta [36]. As a result, no increase in congenital malformations compared to that of the general population was recorded, i.e., 3–4.5% [36]. In line with these data, the EULAR report identified congenital malformations in 3.9% of newborns [37]. Increased spontaneous fetal losses and prematurity (approximately 20 and 30%, respectively), could not be solely attributed to previously administrated tocilizumab, since concomitant methotrexate could have complicated outcomes [36,38]. Among all cases that continued or resumed tocilizumab beyond the first trimester, live infants were born, even though they were preterm in half of the cases [36]. Unfortunately, in reports where patients received tocilizumab beyond the first trimester, outcomes were not reported separately, and hence did not allow for safe conclusions to be drawn [39,40].

Only the RECOVERY trial permitted enrollment of pregnant people (0.2% of participants), while specific maternal and neonatal outcomes were not reported [24]. There have been a number of reports of tocilizumab use in COVID-19 pregnant women [41,42,43,44]. Higher rates of premature death and spontaneous abortion have been reported compared to the general population; however, several confounders do not allow for safe conclusions to be drawn [44]. Most patients were in the third trimester and were critically ill, while concomitant administration of corticosteroids occurred in many cases [41,42,43,44]. Even though all pregnancies resulted in live births, neonatal follow-up was limited, while one case resulted in maternal CMV reactivation and fetus infection [41].

Even though detectable levels of tocilizumab have been recorded in breast milk, especially from mothers of preterm infants, IgG oral bioavailability is low due to degradation in the infant digestive tract [45,46,47,48]. Hence, it seems quite expected that no adverse effects were noted in cases of breastfed infants whose mothers were treated with tocilizumab [40,47,48]. However, data on safety is limited in the second and third trimester, and live vaccines should be delayed to 6 months of age if the infant was exposed to tocilizumab in utero [49].

#### 3.3.3. Baricitinib

Baricitinib represents an orally administered JAK-inhibitor that interrupts the multiple cytokine pathways implicated in COVID-19 immunopathology [50]. It also exerts its action via potential antiviral activity, by blocking viral cell entry and suppressing type I interferon-driven angiotensin–converting-enzyme-2 upregulation [50]. When used in combination with remdesivir, it shortens time to recovery in COVID-19-hospitalized patients, while recently it was found to reduce mortality when added to corticosteroids [51,52]. Pregnant and breastfeeding patients were not included in either of the two trials. No human data exist as to the transplacental passage of baricitinib. However, as a small molecule and in line with animal data, it is expected to extensively cross the human placenta from early on in pregnancy, being teratogenic and feticidal [53].

Clinical data on baricitinib in pregnancy are scarce, mostly deriving from a rheumatoid arthritis clinical trial program [54]. Four of twelve women included had spontaneous fetal loss, one was electively terminated, five had live births with no fetal abnormalities, and two pregnancies were still ongoing by the time this report was filed [54]. In a case report of a woman treated with baricitinib for rheumatoid arthritis from conception to 17 weeks of gestation, a healthy infant was born at 38 weeks, while the baby had normal growth and psychomotor development, and no perinatal infectious occurred at 9 months [55]. No human studies have reported the safety of baricitinib in lactation, but similar to pregnancy, it is likely to be present in breast milk [53]. The thrombotic risk that JAK inhibitors carry is concerning in pregnant people who are already at increased risk of thrombotic complications [50].

#### 3.3.4. Anakinra

Anakinra represents an interleukin-1 (IL-1) receptor antagonist that has recently received FDA approval in COVID-19 treatment following results of SAVE-MORE randomized trial, showing increased benefit among different subpopulation of patients both in short- and long-term outcomes [56,57]. Information related to the use of anakinra during pregnancy and lactation remains limited [58,59,60,61]. Administration of supratherapeutic doses of the drug to pregnant rats and rabbits has not been associated with fetal harm. In a small cohort of 17 patients, the mortality rate was 7.1%, the median hospitalization length was 15 days, while two patients had premature births [62]. Use of anakinra by breastfeeding women has not been associated with adverse effects [63].

### 3.4. Anti SARS-CoV-2 Monoclonal Antibodies

#### Tixagevimab/Cilgavimab

Several antisevere acute respiratory syndrome-coronavirus-2 mAb (anti-SARS-CoV-2 mAb) products directed against the SARS-CoV-2 spike protein have been evaluated for the treatment of COVID-19 [7]. Use of the monoclonal antibody combination tixagevimab-cilgavimab (as preexposure prophylaxis remained, until recently, an option for individuals, including pregnant people, with a moderate to severe immunocompromising condition) may result in a suboptimal immune response for vaccinated people or for those who cannot receive a recommended series of a COVID-19 vaccine because of a severe adverse reaction to the vaccines or their components [6,7]. The safety of the use of anti-SARS-CoV-2 mAbs in pregnant women is not well defined; however, these appear to reduce the risk of severe disease without increasing the risk of significant adverse maternal or perinatal outcomes [64,65]. Human immunoglobulin G1 (IgG1) antibodies are known to cross the placental barrier. Nonclinical reproductive toxicity studies for tixagevimab and cilgavimab have not been conducted, although a tissue cross-reactivity study assessing off-target binding to human fetal tissues found no binding of clinical concern [66]. The phase I NCT05281601 study will investigate safety of intramuscular or intravenous tixagevimab/cilgavimab in patients aged >29 weeks gestational age to <18 years [7]. There are no data on the use of these mAbs in breastfeeding. As IgG1, their transfer into breast milk is expected to be low and they likely undergo degradation in the infant digestive tract; however, this requires confirmation [45,46].

### 3.5. Vaccination

All unvaccinated people planning pregnancy or those who are pregnant or recently pregnant should undergo COVID-19 vaccination—preferably with a nonvector vaccine—and those who are vaccinated should receive booster doses, when eligible, in agreement with major medical organizations and public health authorities [67]. Although fetal and newborn antibody levels appear to be higher with primary vaccination later in pregnancy, early-in-gestation vaccination provided a higher benefit against maternal risk of hospitalization because of COVID-19, death from COVID-19, and COVID-19-related pregnancy complications [68,69]. However, with regard to infants, relating to the risk of hospitalization during the first six months of life, maternal COVID-19 vaccination with a second dose during pregnancy was highly effective against delta and moderately effective against omicron [70]. In a national prospective cohort study from Italy including 2147 women, it was shown that the incidence of moderate or severe COVID-19 during the omicron surge was rare, but was significantly higher in unvaccinated individuals [71].

Prior history of SARS-CoV-2 should not refrain from vaccination. Similar to therapy, pregnant and breastfeeding people were not included in the initial large vaccine trials; however, later data from vaccinated pregnant people showed efficacy before, as well as during pregnancy, postpartum, and during lactation. In a recent metaanalysis, the effectiveness of mRNA vaccination against RT-PCR confirmed that SARS-CoV-2 infection at 7 days following the second dose was 89.5% [72]. The risk of stillbirth was 15% lower in the vaccinated cohort, and there was no evidence of a higher risk of miscarriage, preterm birth, lower birthweight, placental abruption, pulmonary embolism, postpartum hemorrhage, maternal death, or maternal or neonatal intensive care unit admission [72]. Maternal completion of COVID-19 vaccination series during pregnancy was also associated with reduced risk for COVID-19 hospitalization among infants <6 months of age [73], even though, efficacy appears to be lower during circulation of omicron compared to delta surge and higher when the vaccination completion occurred after 20 weeks of gestation compared with before 20 weeks. Population data deriving from Scotland revealed that 77.4% of SARS-CoV-2 infections, 90.9% of SARS-CoV-2 associated with hospital admission, and 98% of SARS-CoV-2 associated with critical care admission, as well as all baby deaths, occurred in pregnant women who were unvaccinated at the time of COVID-19 diagnosis [74].

At the moment, none of the available vaccines recommended for pregnant individuals contain a virus that replicates; hence, they do not cause the disease, but nonetheless nonspecific adverse effects may take place. However, most data derive from mRNA vaccines, and data from early reproductive and toxicity studies have reported similar efficacy and safety [75,76,77]. Rare side effects include thrombosis with thrombocytopenia syndrome, myocarditis, and pericarditis, Guillain–Barré syndrome, as in nonpregnant individuals; however, no infertility issues have been reported [78,79,80,81]. Pregnancy by itself does not represent a cause for increased risks of any adverse outcome, following COVID-19 vaccination [82], while the overall risk of the occurrence of an acute adverse event requiring medical attention is low (<1%) and similar in pregnant and unvaccinated pregnant patients [83]. Direct or indirect harmful effects on fertility, embryo/fetal development, pregnancy outcome, parturition, or short-term postnatal development of offspring were not detected [75,84,85,86,87,88,89,90,91,92,93,94,95,96,97]. A recent metaanalysis did not find significant differences in assisted reproductive outcomes between vaccinated and unvaccinated women [98]. The minimal amount of vaccine that is detected in breast milk and ingested by the infant is likely to be inactivated by the infant’s digestive system, and hence unlikely to pose risks to the infant’s health [99,100]. However, clinical trials did not include the breastfeeding population. In summary, vaccination in the pregnant population is effective and safe; however, as expected at the moment only data from observational studies are available, while even within this target group the population remains diverse [101].

### 3.6. Study Limitations

This was a literature review regarding pregnant patients’ experience with COVID-19 pharmacotherapy throughout the last three years of the SARS-CoV-2 pandemic. This review bears a number of limitations. Even though a systematic and transparent description of methods is presented in the respective sections and Figure 1, bias in the selection and interpretation of studies is possible, as in every literature review. As commonly occurs in the literature, data pertaining to pharmacotherapy and pregnancy, even during COVID-19 pandemic, remain scarce. A lack of randomized clinical trials and drug testing in this population only allows for extrapolation of conclusions from experimental models and real-world data. Moreover, the authors carried out a literature review in a rapidly evolving field that has utterly changed since the beginning of the pandemic. It is possible that by the time this manuscript is published, therapeutic regimens may be outdated or more data is available. The use of tixagevimab/cilgavimab, which has now been withdrawn due to circulating variants, is an example. Nonetheless, the authors have chosen to include it in this study during revision for proof of concept. In this expanding field, data remained equally diverse and mixed during different waves and/or variant surges not allowing for further temporal analysis, even though drawbacks mainly exist in prevention regimens, and are discussed in context. In addition, only English-language papers were reviewed. Experience recorded in other languages contributing significant input may have been missed. Similarly, data and outcomes from vaccine rollout programs in pregnant populations in low- or middle-income countries may not be included in this review due to severe disparities in health policies and population hesitancy [102,103]. Moreover, one cannot overcome potential publication bias that already excludes negative results. Grey or unpublished literature was not assessed in this report. Lastly, the aim of this review was to record issues relating to antiviral and immunomodulating issues pertaining to COVID-19 pharmacotherapy. No information is reported regarding adjunctive or supporting care, e.g., anticoagulants [104], etc.

## 4. Conclusions

Pregnant people should be counseled about the increased risk for severe disease from SARS-CoV-2 infection and receive recommendations on ways to protect themselves and their families from infection. Potentially effective treatments for COVID-19 should not be withheld from pregnant people because of theoretical concerns related to the safety of using those therapeutic agents in pregnancy [6]. The lack of high-quality data on the use of COVID-19 pharmacotherapy during pregnancy and breastfeeding highlights the need for a more significant regulatory push and incentives to accelerate studies obtaining pregnancy data. Pediatric licensing trials have been successful in providing valuable data on the safety and efficacy of drugs in children, and a similar approach is needed for drugs used in pregnant and breastfeeding individuals. These studies are crucial to inform evidence-based decisions and optimize outcomes for both the mother and the infant. Regulatory agencies can play a key role in encouraging the inclusion of pregnant and breastfeeding individuals in clinical trials. For example, the FDA has issued guidelines for the inclusion of pregnant and breastfeeding individuals in clinical trials, and the European Medicines Agency (EMA) has established a Pregnancy and Breastfeeding Taskforce to provide guidance on drug use during pregnancy and lactation. Incentives, such as priority review or market exclusivity, can also encourage drug companies to conduct studies in pregnant and breastfeeding individuals. Table 1 provides concise information on the drugs discussed, along with current recommendations. More high-quality data is needed to ensure the best outcomes for patients with COVID-19 who are pregnant or breastfeeding. This includes data on the safety and efficacy of existing COVID-19 pharmacotherapies in this population, as well as the development of new drugs specifically for use during pregnancy and lactation. In the absence of high-quality data, clinicians must make treatment decisions based on the available evidence and individual patient factors. However, the lack of data can lead to uncertainty and variability in clinical practice, highlighting the urgent need for more studies in this population.

## Figures and Tables

**Table 1:** COVID-19 pharmacotherapy in pregnancy: Summary of data.

	Efficacy	Safety	Current Recommendations
Antiviral therapy			
Remdesivir	Early administration in COVID-19 pregnancy was associated with Improved clinical outcomes including higher recovery rate, decreased ICU admissions and progression to critical disease Improved pregnancy outcomes including decreased maternal deaths from COVID-19 and lower rates of preterm delivery	No obstetric indications for preterm delivery or neonatal deaths were noted There is lack of data on lactation, but no adverse effects are expected due to low oral bioavailability	Recommended
Nirmatrelvir/ritonavir	Outpatient treatment in COVID-19 pregnancy resulted in increased recovery According to ACOG and the Society of Maternal Fetal Medicine, the benefits outweigh the risks	It is considered safe No data are available for nirmatrelvir, while limited data suggest the presence of ritonavir in breast milk No information on breastfeeding infants or milk production Lactation should continue with monitoring for infant side effects	Recommended
Molnupiravir	It has shown antiviral activity against SARS-CoV-2 in vitro and in clinical trials Lower efficacy than the other approved antiviral agents No human clinical data in pregnancy	Fetal toxicity has been reported in animal studies Lactating people should not breastfeed until four days after the final dose	Not recommended, unless there are no other options and therapy is clearly indicated
Immunomodulation			
Corticosteroids	Dexamethasone decreases mortality in individuals with COVID-19 in need of oxygen supplementation	It has been associated with adverse pregnancy outcomes including congenital malformations, intrauterine growth restriction, gestational diabetes, preterm birth, and preeclampsia Low amount of dexamethasone present in breast milk High doses may temporarily decrease milk production	Recommended (hydrocortisone, methylprednisolone, prednisolone, and prednisone preferred to dexamethasone)
Tocilizumab	Mortality reduction in hospitalized adults with severe or critical COVID-19 Limited human cases in otherwise critical COVID-19 pregnant patients	Transplacental transport is thought to be very low during the first semester and then increase steadily No new congenital malformations were recorded Preterm births and spontaneous abortions in presence of confounders, e.g., critical disease Detectable amounts in breast milk, but no adverse effects are recorded due to low oral bioavailability Live vaccines should be delayed to 6 months of age	Recommended
Baricitinib	Faster recovery when used in combination with remdesivir Reduced mortality when added to corticosteroids No data in human COVID-19 pregnancy	There is no data for human transplacental transport. In line with animal data, it is expected to cross the human placenta and cause teratogenesis or fetal death No human studies available for lactating individuals, but its presence in breastmilk is expected As a JAK inhibitor, it shows an increased thrombotic risk	Recommended
Anakinra	Improved clinical outcomes both in short and long term Limited human data in COVID-19 pregnancy, in combination with other regimens	Supratherapeutic doses on animal studies showed no fetal harm No adverse effects reported when used by breastfeeding women	Insufficient evidence to recommend for or against
Prevention			
Vaccination *	Similar immunogenicity to nonpregnant individuals and independent of trimester of vaccination Protected against maternal severe disease and infant hospitalization during the first 6 months of life	Similar rates with nonpregnant population No safety signals among obstetric or neonatal outcomes including rates of pregnancy loss, preterm birth or congenital anomalies	Recommended
Monoclonals (Tixagevimab-cilgavimab)	Until recently, tixagevimab–cilgavimab was used as preexposure prophylaxis for individuals, including pregnant women, with suboptimal response to vaccination due to an immunocompromising condition or who are unable to vaccinate for COVID-19 because of adverse effects There was marked reduction in the in vitro effectiveness against the current omicron variants	The safety is not well defined There was no additional risk of significant adverse maternal or perinatal outcomes Transplacental transport is likely, but no significant fetal cross-reactivity binding was recorded There is lack of data on lactation, but no adverse effects are expected due to low oral bioavailability	Recommended against (due to currently circulating variants)

* Data mainly deriving from mRNA vaccines.

## Data Availability

Not applicable.
